# Be in Your Element: The Joint Effect of Human Resource Management Strength and Proactive Personality on Employee Creativity

**DOI:** 10.3389/fpsyg.2022.851539

**Published:** 2022-03-29

**Authors:** Jiexuan Zhang, Fei Zhu, Ning Liu, Zijun Cai

**Affiliations:** ^1^Business School, Central University of Finance and Economics, Beijing, China; ^2^Business School, Beijing Normal University, Beijing, China

**Keywords:** human resource management strength, job crafting, career adaptability, proactive personality, creativity

## Abstract

Employee creativity is fast becoming a part and parcel in the wake of the increasing volatility of the employment market and the complexity of job demands. Drawing from the actor-context interactionist theoretical approach and career construction theory, this paper adds to current research by exploring the serial mediating effect of job crafting (JC) and career adaptability (CA) in the impact of human resource management strength (HRMS) on employee creativity. Furthermore, we suggest that proactive personality interacts with HRMS to jointly influence creativity. Survey data from samples of 297 (Study 1) and 390 (Study 2) employees largely confirm our model. Our findings show that HRMS positively impacts employee creativity *via* serial mediation of job crafting and career adaptability, and proactive personality negatively moderates the process. The paper confirms and expands the interactionist theoretical perspective of creativity, highlights the significance of integration of contextual factors, individual characteristics, and career construction, and makes certain practical sense.

## Introduction

Employee creativity has become a significant factor impacting organizational efficiency and sustainable competitive advantages. To this end, organizations highlight factors that are likely to boost individual creativity by putting at their disposal appropriate systems (Moussa and El Arbi, 2020). In a similar vein, an increasing amount of research started focusing on determining how general contextual factors inspire or inhibit employee creativity, and how different perceivers respond to context (Zhou et al., 2017). From the perspective of context-centered approach (Zhou and Hoever, 2014), employee creativity highly depends on organizational contexts, such as leadership supervision (Liu et al., 2012), leadership style (Gong et al., 2009), and the value and culture of uncertainty avoidance and justice (Shalley and Gilson, 2004). Since individuals first appraise features of HR system as positive, neutral, or stressful at work (Johnston, 2018), HR practices (i.e., selection, training, evaluation, and rewards; Shalley and Gilson, 2004; Yasir and Majid, 2020) are crucial at fostering innovation processes in companies by influencing creativity (Acosta-Prado et al., 2020). Although scholars have emphasized different characteristics (Shalley and Gilson, 2004) and outcomes (Yasir and Majid, 2020) while describing HRM systems, the most significant characteristic is human resource management strength (HRMS; that is distinctiveness, consensus, and consistency of HR practices), and the most exciting outcome is at the individual level, i.e., employee creativity. However, research on the potential influence of HRM systems on employee creativity is limited (Rehman et al., 2019).

Human resource management systems are crucial but not sufficient to affect employee creativity directly, there is a need to understand the internal mechanism that might be involved in this relationship (Yasir and Majid, 2020). Creativity is also the function of the interplay between multiple actor-level variables, as well as creativity-related behaviors (Zhou and Hoever, 2014). Additionally, the current paper pays more attention to the ability and resource of career construction, namely career adaptability (CA). CA is self-regulation strength or capacity (Savickas and Porfeli, 2012), guiding individuals to prepare and replenish resources for coping with current and imminent vocational development tasks, occupational transitions, and personal traumas (Savickas, 1997, 2005; Creed et al., 2009; Johnston, 2018), to achieve the goal of person-environment integration in the career transition (Savickas and Porfeli, 2012; Pan et al., 2018). CA is a malleable resource (Akkermans et al., 2020) and is developed through various personal experiences (Savickas, 2013) and behaviors (Guan and Frenkel, 2018; Chen et al., 2020), such as career exploration (Cai et al., 2015; Guan et al., 2015; Li et al., 2021), training activities, and other activities designed to help one update existing knowledge and better adapt to changing environments [i.e., job crafting (JC); Mei et al., 2021]. JC is a bottom-up and repetitive work redesign process, which is perceived as an important factor in overcoming the uncertain and rapidly changing work environment and provoking CA (Woo, 2020). It entails approaching and avoiding the needs of job resources and roles to avoid situational threats, as well as actively seeking and acquiring resources to expand work content or roles (Bruning and Campion, 2018). However, not much work has been done to examine the effects of behaviors on career adaptability (Guan et al., 2015).

Apart from the ability of career construction, individuals differ in their willingness or readiness to affect change (Savickas and Porfeli, 2012). Intrinsic motivation and creative personalities (Zhou, 2003) also prove to be factors with a significant effect on creativity (Moussa and El Arbi, 2020). Proactive personality reflects one’s intrinsic dispositional behavioral tendency and action orientation to effectively achieve personal goals (Cai et al., 2015) and urges individuals to scan for opportunities, show initiative, take action, and persevere until they reach closure by bringing about change (Bateman and Crant, 1993). Nonetheless, it is not clear whether and how proactive personality may contribute to creativity in the context of uncertainty (Yi-Feng Chen et al., 2021).

**Figure 1:**
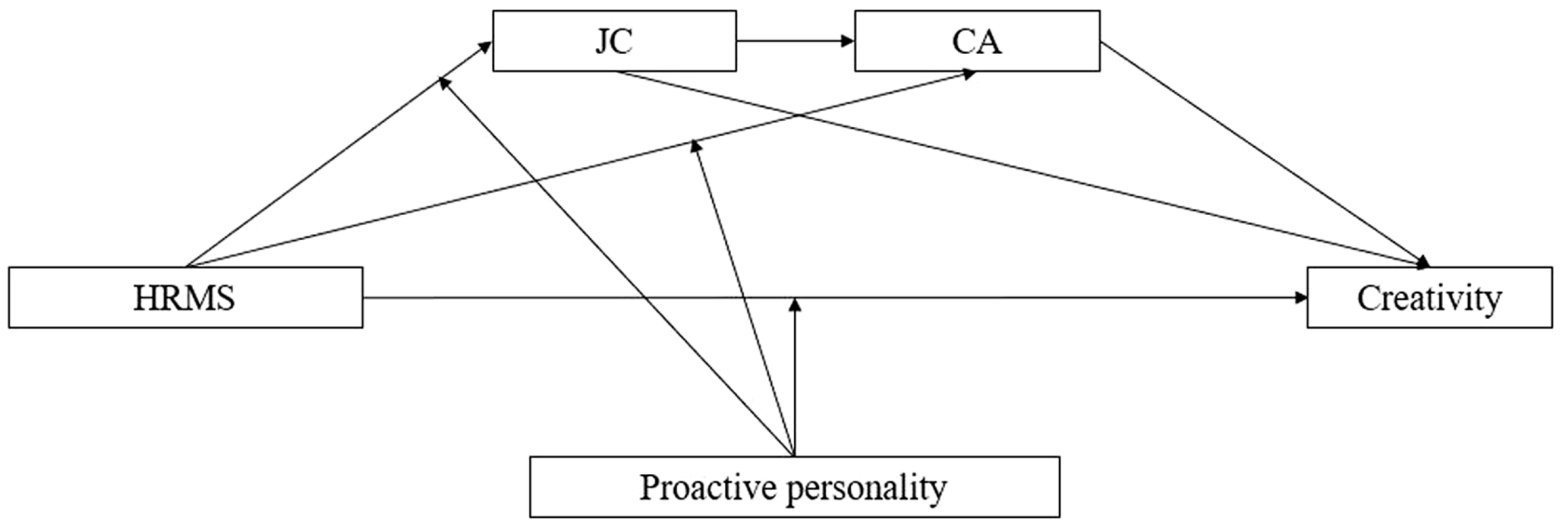
Theoretical model. HRMS, human resource management; JC, job crafting; and CA, career adaptability.

In summary, the main issues addressed in this paper are (a) the impact of HRMS on employee creativity, as well as the serial mediation of JC and CA in the process, (b) the moderating role of proactive personality in the process of HRMS influencing employee creativity through JC and *CA.* We do so by examining through which mechanisms (Study 1) and under which conditions (Study 2) HRMS are related to creativity. An overview of our theoretical model can be found in Figure 1.

This paper makes several contributions to the literature. First, under the condition of fierce competition, environmental uncertainty, and instability, the current paper focuses on employee creativity, confirms the actor-context interactionist theoretical perspective, and expands the antecedents of creativity, namely behaviors and abilities. Second, we discuss employee creativity from the perspective of career construction, synthesize adapting strategy (JC) and adaptability (CA) simultaneously and provide an exhaustive perspective of career development for researchers and practitioners. Third, this paper explores the joint effect of contextual factors and individual characteristics on the construction of careers and provides a comprehensive explanation. Specifically, we challenge the universal standpoint that proactive personality is always positive, providing empirical and theoretical evidence for the duality of proactive personality, which means that the efficiency of HR practice will be mitigated by the proactivity of employees, supporting existing research (George and Zhou, 2001; De Dreu and Weingart, 2003; Jimmieson et al., 2004; Spychala and Sonnentag, 2011).

## Study 1: The Influence of Organizational Context on Employee Creativity: The Serial Mediation of Job Crafting and Career Adaptability

### Literature Review and Research Hypothesis

#### Human Resource Management Strength and Employee Creativity

In emerging economies that advocate technological innovation to promote social and economic development, a vital development trend in global strategic HRM is the design and implementation of HR systems that can boost employee initiative and creativity (Liu et al., 2017). Creativity refers to the ideas considered as original, unique, or unconventional by normative standards (Zhou et al., 2017) in the process of career construction, and it means more appropriate, flexible, and innovative ideas about work roles and methods (Maggiori et al., 2013), intending to improve work flexibility and productivity (Scott and Bruce, 1994). Given the dynamic nature of the context (Savickas and Porfeli, 2012), employee creativity varies as a function of the characteristics of the task and work, as well as constantly changing professional situations (Maggiori et al., 2013; Johnston, 2018), such as the aspects of the task, the physical environment, and the social environment (Zhou and Hoever, 2014). A literature review adopting scientific knowledge mapping has shown that the study of contextual factors on career responses is mainly focused on social support (Chen et al., 2020). In recent years, there has been an increasing amount of literature on the promotion or inhibition of HR practices on career behaviors (Zhou and Hoever, 2014; Liu et al., 2017; Zhou et al., 2017). HR practices boost employee creativity by developing the required knowledge, skills, and abilities, which are important for the execution of discretionary and extra-role activities, enhancing employees’ motivation to engage in idea creation and testing these ideas, providing opportunities and execution of developing skills and behavior at the workplace (Rehman et al., 2019; Yasir and Majid, 2020). The key is to provide employees with an environment that is challenging enough but not so overstimulating that employees feel overwhelmed and unable to break out of old ways of doing their work (Joo et al., 2013), as an important characteristic of an organizational HR system, HRMS reflects dimensions of the work environment that potentially influence an employee’s creativity (Shalley and Gilson, 2004).

Human resource management strength is perceived in three aspects: distinctiveness, consistency, and consensus (Bowen and Ostroff, 2004; Ostroff and Bowen, 2016). Distinctiveness refers to the attention, interest, and attention of individuals in the context; consistency refers to the behaviors are presented in a special way that is expected. This transmission process is the same in both the global and local parts of the organization and is consistent with the organization’s goals; consensus emphasizes the unanimous recognition of the content of HR practices by employees (Bowen and Ostroff, 2004). When the decision-makers are generally aware of certain information, the distinctiveness is strengthened, thereby promoting employee consensus and clarifying the strategic direction of the organization, or else it is difficult to send clear communication and form internally consistent information. Therefore, we believe HRMS can play a pivotal role in enhancing employee creativity and in building a more appropriate and supportive contextual environment for creativity (Joo et al., 2013) by providing employees with a similar “cognitive map” and an “impact context” that can reduce uncertainty among employees by strengthening the clarity of information transmitted (Bowen and Ostroff, 2004). According to career construction theory (CCT), the dynamic characteristics of the context are highly correlated with individual career outcomes (such as creativity; Savickas and Porfeli, 2012), and earlier research has also confirmed that HRMS has an impact on employees’ improvised behaviors, i.e., employee creativity (Pereira and Gomes, 2012).

The distinctiveness, consistency, and consensus of HRMS facilitate employee creativity. Firstly, the distinctiveness and consensus of HRMS mean a more certain and stable organizational context (Pereira and Gomes, 2012). Compared with the context where the expectations and boundaries are not clear, individuals can enjoy more mental resources to experience cognitive flexibility and creativity in a clear and certain context (Zhang and Zhou, 2014). At the same time, creativity in the workplace is not aimless brainstorming, and we must first be sensitive to the rules, guidelines, and constraints in the organization, that is, the accuracy of ‘inside the box’ cognition can further improve the effectiveness of “outside the box” creative thinking (Zhang and Zhou, 2014). Uncertainty avoidance employees often have a more thorough understanding of rules, guidelines, and constraints, so HRMS ensures that employees can bring not only novel but also practical innovation (Zhang and Zhou, 2014).

Second, the consistency of HRMS can enhance the sense of organizational support for employees (Stanton et al., 2010). When leaders, policy implementers, and employees have consistent understanding and implementation of organizational HRM information, leaders at different levels can jointly support their subordinates, and give them consistent guidance, help, and authorization, with consistent understanding, support, and encouragement, employees can be more interested and focused on their work tasks instead of unnecessary worry and fear, so they can be more venturesome, unrestrained to explore through trials and errors (Shin and Zhou, 2003), which are key to translate to creativity. Consistency is also conducive to promoting effective communication so that employees can have a deeper understanding of the work duties. In another word, efficient communication channels ensure the dissemination, recognition, and practice of innovative thinking, and help people relate to the organization and strengthen their motivations and behaviors of being creative (Scott and Bruce, 1994; George and Zhou, 2001). Based on the above theoretical analysis, we propose that:

*H1: HRMS positively impacts employee creativity.*

#### The Mediating Effect of Job Crafting and Career Adaptability

Career construction theory incorporates and updates previous theoretical contributions and frameworks (such as career development theory, developmental self-concept theory, life-span, and life-space theory), tightens the integration between the life-span, life-space, and self-concept segments by focusing each on the individual’s adaptation to environmental context and emphasizing a single source of motivation (Savickas, 1997). CCT believes that individuals must fully self-direct and construct in the process of career development to adapt to changes in work content and methods, and to prepare for more professional tasks, roles, and opportunities (Hall, 2002). There are three major components in the process of constructing a career, namely vocational personality (individual personality characteristics), career adaptability (coping styles and processes), and life themes (career development models). CCT clarifies individual personality differences, coping strategies, psychological motivations, and development tasks (i.e., the content, methods, and concept orientation of career development) in the construction of careers from the perspectives of society (i.e., social expectations) and individuals (i.e., how individuals respond to social expectations; Savickas, 2005).

Job crafting is a proactive behavior and adapting the strategy of employees, which refers to changes in work tasks and work relationships, to make their work better meet their personal needs, goals, skills, values, or interests (Berg et al., 2010; Bipp and Demerouti, 2015), compromised of structure (i.e., work content and procedures), social cognitive forms, and physiology (Wrzesniewski and Dutton, 2001; Bruning and Campion, 2018; Guan and Frenkel, 2018). It is a dynamic process of continuous adjustment and change to improve the fit between individuals and the environment (Parker and Collins, 2010; Wang et al., 2017; Federici et al., 2021). Derived from inner drives and personal will, with an aim of self-development, it often leads to remarkable improvements in work content, social activities, and cognition, and most of such improvement occurs in roles and work based on clear descriptions and specified tasks (Bruning and Campion, 2018). The motivation for JC can be either active or passive (Lazazzara et al., 2020), but they all reflect a tendency to improve the match between job characteristics and personal needs, abilities, and preferences to cope with continuous environmental changes (Zhang et al., 2019), which not only expresses self-concept, but also integrates with social context and work context to help individuals create deeper and broader meaning in their daily work, and find better ways to overcome social barriers (Savickas, 2005).

Contextual factors are ultimately the core linking personal motivation with different forms of JC (Lazazzara et al., 2020), and play an important role in promoting employee JC. Existing research confirms the impacts of person-job fit (Tims and Bakker, 2010), organizational identification (Lazazzara et al., 2020), and perceived organizational support (Kim et al., 2018) on JC. In recent years, the impact of HRMS on JC has also gradually been in the limelight. It has conclusively shown that a strong HRMS can increase job resources and reduce hindrance of job demands to achieve higher performance and creativity (Guan and Frenkel, 2018). In a similar vein, earlier research identifies five core job characteristics, including skill variety, task identity, task significance, autonomy, and feedback (Hackman and Oldham, 1980), that enhance employees’ internal motivation, attitudes, and eventually help facilitate positive work outcomes and creative performance at work (Lee and Lee, 2018). Therefore, changes in the HRMS can promote task significance and identity, in turn, promotes employee creativity. First of all, when HR policies and practices are unique, employees are often able to identify and actively reflect on organizational policy practices (Bednall et al., 2014), thereby learning, adjusting work tasks through reflection, improving work efficiency, and participating in more complex and challenging tasks. In addition, when there is an agreement on HR practice among HR policymakers and supervisors, employees are less likely to experience role ambiguity and task conflict (Bowen and Ostroff, 2004). They will have a better understanding of management intentions, expectations (Guan and Frenkel, 2018) and be more able to utilize resources efficiently and reduce the requirements that bring obstacles to work. Existing studies have also confirmed that committed HR practices are positively related to JC (Hu et al., 2020), because the goal of committed HR practices is to align employees with organizational goals and commit to each other, making employees more proactive and continue to engage, where employees meet job demands through JC reinvest and maintain the large number of resources given by the organization (Meijerink et al., 2020).

Career construction theory believes that individuals need to develop self-regulation resources to achieve the goal of person-environment integration in various career transitions (Pan et al., 2018; Yu et al., 2019), adjust work requirements and work resources, improve work efficiency, and expand work results, JC is the self-development, actively used, and continuous control of resources. Therefore, it will produce an incentive process that promotes career success (Akkermans and Tims, 2017; Mei et al., 2021). On the one hand, through bottom-up JC (van de Riet et al., 2015), employees will engage in achieving work goals and generating and implementing new ideas by using abundant resources (Afsar et al., 2019). On the other hand, the process of job redesigning can improve the match between the individual and the organization (Tims and Bakker, 2010), making them more confident and motivated. JC facilitates the process of creating change as employees feel energetic, enthusiastic and engaged in challenging the status quo (Afsar et al., 2019; Sun et al., 2020), thereby not only efficiently completing the goals and meeting performance requirements specified by organizations but also promoting extra-role behaviors, such as creativity (Demerouti et al., 2015; Gordon et al., 2015; van de Riet et al., 2015; Lee and Lee, 2018). Therefore, we propose the following hypothesis:

*H2: Job crafting mediates the relationship between human resource management strength and employee creativity.*

The career construction process underlines the relevance of the interaction between the individuals and the environment, as well as the adaptability to cope with novel and unprecedented issues (Creed et al., 2009). CA is self-regulation strength or capacity. Career adaptability oozes the appeal from employees to better acclimatize to the environment (Savickas and Porfeli, 2012; Chen et al., 2020), it is a malleable psychosocial resource for individuals to manage the current and prospective changes in their job roles caused by changes in work content and work conditions, and can transform the process of social integration of employees, including the career development, selection, and adjustment (Savickas, 1997, 2005). It can also be perceived as an emotional trend when employees view their own script or adjust the shifting sands of their career planning, especially in the face of unforeseen events (Rottinghaus et al., 2005).

Career adaptability resources have an intervening effect on perceived contextual factors. Previous empirical research shows that CA partially mediates the relationship between work conditions (including job strain and professional insecurity) and well-being (Maggiori et al., 2013). We argue that HRMS is positively related to CA for two reasons. First, job insecurity and strain can hinder employees’ career planning, leading to a drain on career adaptability resources and responses (Klehe et al., 2011), such as self-regulate ability, adaptability, and self-awareness. As far as HRMS is concerned, on one hand, employees’ perception and attitudes toward HR practices will affect the willingness of human capital investment and accumulation amid their career construction (Chen et al., 2007). Second, HRMS reduces insecurity and job strain within the organization by increasing the quantity and concentration of internal communication channels, networks, and content in the organization (Ostroff and Bowen, 2016), thereby improving employees’ professional resilience resources and coping capabilities. Therefore, it is necessary for employees to customize personal resources adaptable to the HRM system to improve compatibility.

Cognitive style and ability is a key antecedent of creativity, such as problem finding, problem construction, combination, and idea evaluation are important for creativity (Shalley and Gilson, 2004). CCT believes that career adaptability includes four dimensions, namely career concern (presuming, planning, and preparing for the possibility of future development), career control (prudent decision-making and serious action and shaping the personal responsibility of the future), career curiosity (exploring various possible roles and selves), and career confidence (the belief that individuals can make choices and achieve goals when dealing with obstacles and problems; Savickas, 2005; Johnston, 2018). According to CCT, the employees’ concern for career development nudges them into considering and exploring various possible situations in the future, and making them prepared, while career control ensures the feasibility and effectiveness of exploration and innovative behaviors and shifts employees toward making decisions and acting seriously based on “in-the-box” thinking. Career curiosity in career development is equivalent to the expansion of work tasks and roles (Johnston, 2018). Finally, career confidence ensures the employees’ continuous motivation in their innovative thinking of work problems and obstacles. Adaptable employees do not limit their efforts to meet the requirements of the assigned tasks and official targets, they also have sufficient capabilities and resources to broaden task boundaries and engage in extra-role behaviors (Lan and Chen, 2020; i.e., creativity). Therefore, employees with strong career adaptability will actively engage in career creativity. In other words, we propose that career adaptability will mediate the HRMS-creativity relationship beyond the mediation of JC:

*H3: Career adaptability mediates the relationship between human resource management and employee creativity.*

Adaptability as a psychosocial resource or transactional competency is more changeable than traits (Savickas and Porfeli, 2012). CA has plasticity, as well as some continuity in choices and adjustment, and is trainable and open to development (Vogt et al., 2015), with the changes in individual life and working environment, self-concept, occupational preferences, and occupational adaptability will change along with time and experience (Savickas, 2005). Career construction is prompted by vocational development tasks, occupational transitions, and personal traumas, and then generated by the reaction to these career changes (Savickas, 2005). A minicycle of growth, exploration, establishment, management, and disengagement occur during transitions from one career stage to the next as well as each time an individual’s career, which eventually forms the entire career (Savickas, 2005).

Employee creativity and other professional behaviors must not only take current JC into account but also long-term career development (Woo, 2020). JC refers to self-initiated behaviors that may help individuals to deal with these changes (Demerouti et al., 2017), involves the immediate adjustment of resources, and needs related to tasks or roles in the current job. It is a short-term and repetitive solution (Tims and Bakker, 2010), and an improvised, creative process (van de Riet et al., 2015). In the early stage of career construction, employees are more likely to promote job development by increasing challenging job demands. Expansive JC in terms of increasing job resources and challenging job demands should stimulate personal growth, development, and adaptability (Tims and Bakker, 2010; Maggiori et al., 2013; Akkermans and Tims, 2017) and help employees establish a more positive and stable emotional status at work (Xu et al., 2020), such as *CA.* CA is a cumulative outcome, a consequence of behaviors being amplified over a relatively long period (Seibert et al., 1999), previous studies have suggested that specific exercises and deliberate practice serve as important learning behaviors in the adaptation process (Mei et al., 2021). A higher-level adjustment beyond the current job content, focusing on the needs of long-term career development, considering the development of new and more job-related skills, or exploring different tasks and roles that are more suitable for an individual’s professional abilities (Zhang et al., 2019).

Job crafting is conceptually related to *CA.* Through redesigning structure, social cognitive forms, and physiology of job, one could seek extra resources and challenges, in turn, accumulate more cognitive and practical experiences related to his/her targeted occupations such that they would have confidence and a sense of control in dealing with related problems, and can better plan for the future in relation to careers (Cai et al., 2015; Guan et al., 2015), that is to say, employees to adjust their work to their inner tendencies and to find meaning in their work, which is particularly important for adapting to new realities (Peeters et al., 2016). Construction provides opportunities, elicits motivation, boosts personal adaptability, and improves employee quality, learning ability, and helps with achieving professional goals (Akkermans and Tims, 2017). Empirical research also shows that JC is positively related to personal resources (Wang et al., 2017), because the key steps in JC—setting goals and finding ways to achieve them—are crucial to the development of personal resources (Vogt et al., 2015), and CA is an individual psychological and social resource. We propose hypothesis H_4_ based on the demonstration:

*H4: Job crafting and career adaptability play a serial mediating role in the influence of human resource management strength on employee creativity.*

### Materials and Methods

To test the serial mediating effects of the influence of HRMS on employee creativity, especially to test the effects of JC on CA, this study, based on CCT, collects data from employees at all levels in various industries at three points, and the industry variables include finance, manufacturing, education, agriculture, forestry, animal husbandry, sideline fishery, etc. The professional level includes senior managers, middle managers, frontline managers, and frontline employees to enhance the universality of the research.

#### Data Collection

In order to reduce the common method variance and ensure the validity of the test, the study uses an online questionnaire survey method to collect data, and measures four variables at three time points: measuring the demographic characteristics of employees (control variables) and HRMS at time 1; measuring JC at time 2; and measuring CA and employee creativity at time 3. We collected 399 questionnaires from different industries at time 1. At time, 2, 334 were returned. At time, 3, 297 questionnaires were collected with the overall response rate of the questionnaire being 74.4%. Of the initial cohort of 297 samples, in terms of gender, males accounted for 48.8% and females 51.2%.

#### Measures

The scales used in the questionnaire survey in this study are all derived from existing research and developed scales. The responses for all the items were obtained on a five-point Likert scale (1 = completely disagree, 5 = completely agree). According to existing research tests, the validity of each scale is good.

##### Human Resource Management Strength

We adopt the scale developed by Delmotte et al. (2012). The scale includes three dimensions: uniqueness, consistency, and consensus, and a total of 31 items. For example, “Human resource management in a company is established by consensus between human resource management and front-line management,” “The human resource information is consistent with the words and deeds of the human resources department.” Cronbach’s *α* is 0.929.

##### Job Crafting

The scale adopts the scale developed by Bruning and Campion (2018). The scale includes four dimensions: increasing work roles, reducing work roles, increasing work resources, and reducing work resources, with a total of 30 items, for example, “Proactively express opinions on important issues in order to broaden my work role,” “Proactively expand the scope of work to ensure the smooth progress of my work.” Cronbach’s *α* is 0.762.

##### Career Adaptability

The scale is adapted from the scale developed by Savickas and Porfeli (2012). The scale includes four dimensions of concern, control, curiosity, and confidence, with a total of 24 items, for example, “Think clearly about what my future is it like,” “Keep positive and optimistic.” Cronbach’s *α* is 0.892.

##### Creativity

The scale adopts the scale developed by Tierney et al. (1999). The scale has five items, for example, “Good at discovering new effects of existing working methods or tools,” “Trying to find a new way or way to solve the problem.” Cronbach’s *α* is 0.826.

##### Control Variables

According to previous research, we controlled for gender, age, individual tenure, and job level (1 = senior manager, 2 = middle manager, 3 = frontline manager, and 4 = normal employees), which has been found to distinguish employee creativity (Shin and Zhou, 2003; Hirst et al., 2009; Hennessey and Amabile, 2010) and interact with contextual cues, there is no strong evidence to imply that the industry and the level of the organization would influence interested variables, thus we did not control them.

#### Measurement Models

We use confirmatory factor analysis (CFA) to test the possible common method variance of the main variables (HRMS, JC, CA, and creativity). The results are shown in Table 1. The degree of fitting of five-factor model (χ^2^/*df* = 1.210, RMSEA = 0.027, NFI = 0.994, RFI = 0.982, IFI = 0.999, CFI = 0.999, and GFI = 0.996) is better than other alternative models. In summary, the results measured by this scale are relatively ideal. There is no significant common method variance in this study.

**Table 1:** Confirmatory factor analysis.

Model	*χ*^2^/*df*	RMSEA	NFI	RFI	IFI	CFI	GFI
One-factor model (HRMS +JC + CA + C)	40.068	0.363	0.497	0.396	0.503	0.502	0.739
Two-factor model (HRMS + JC; CA + C)	14.489	0.213	0.891	0.782	0.898	0.897	0.934
Three-factor model (HRMS; JC; CA + C)	1.568	0.044	0.996	0.976	0.999	0.999	0.997
Four-factor model (HRMS; JC; CA; C)	1.210	0.027	0.994	0.982	0.999	0.999	0.996

HRMS refers to human resource management, JC refers to job crafting, CA refers to career adaptability, and C refers to creativity.

### Results

#### Correlation Analysis

Pearson’s correlation analysis was used to test the correlation between the variables, and the results are shown in Table 2.

**Table 2:** Correlation analysis.

Variables	Mean	SD	1	2	3	4	5	6	7	8
1. Gender	1.509	0.501	1							
2. Age	3.472	0.675	0.114^*^	1						
3. Tenure	3.549	1.026	−0.173^*^	−0.668^**^	1					
4. Job level	3.065	0.903	0.054	−0.213^**^	0.302^**^	1				
5. HRMS	3.865	0.474	−0.098	0.028	0.110^*^	−0.169^**^	1			
6. JC	3.636	0.265	−0.091	0.055	0.039	−0.212^**^	0.462^***^	1		
7. CA	4.266	0.347	−0.043	0.027	0.103	−0.151^**^	0.509^***^	0.567^***^	1	
8. Creativity	3.952	0.613	−0.047	0.067	−0.017	−0.196^**^	0.438^***^	0.555^***^	0.607^***^	1

* *p* < 0.05;

** *p* < 0.01; and

*** *p* < 0.001.

It can be seen from Table 2 that HRMS is positively correlated with JC (*r* = 0.462, *p* < 0.001); CA is positively correlated with HRMS (*r* = 0.509, *p* < 0.001), and is positively correlated with JC (*r* = 0.567, *p* < 0.001); employee creativity is positively correlated with HRMS (*r* = 0.438, *p* < 0.001), JC (*r* = 0.555, *p* < 0.001), and CA (*r* = 0.607, *p* < 0.001). Job level is negatively correlated with HRMS (*r* = −0.169, *p* < 0.001), JC (*r* = −0.212, *p* < 0.001), CA (*r* = −0.151, *p* < 0.001), and employee creativity (*r* = −0.196, *p* < 0.001), that is, the lower the job level, the lower the level of the above variables. This result is the basis for subsequent hypothesis testing.

#### Hypothesis Test

In order to test the impact path of HRMS on employee creativity through the serial mediation of JC and CA, all analyses were carried out using SPSS 22.0 to conduct hierarchical regression analysis, the results are shown in Table 3. The models use employee creativity as the dependent variable; models 1–2 add control variables and HRMS. The results show that the model *R^2^* increases by 0.165 after adding HRMS, and is significant at the level of 0.001, HRMS significantly positively affects the level of creativity (*β* = 0.547, *p* < 0.001).

**Table 3:** The mediating effect test.

	Model 1	Model 2	Model 3	Model 4
Intercept	4.261^∗∗∗^	2.216^∗∗∗^	−0.765	−1.466^∗∗^
Gender	−0.058	−0.019	0.016	−0.002
Age	0.082	0.031	0.021	−0.008
Tenure	−0.013	−0.040	−0.038	−0.066
Job level	−0.149^∗∗∗^	−0.106^∗∗^	−0.063	−0.064
HRMS		0.547^∗∗∗^	0.289^∗∗∗^	0.129
JC			1.052^∗∗∗^	0.642^∗∗∗^
CA				0.714^∗∗∗^
*R^2^*	0.052	0.216	0.358	0.442
*R^2^*	0.052	0.165	0.142	0.097
*F*	3.975^∗∗^	16.063^∗∗∗^	26.928^∗∗∗^	34.444^∗∗∗^

*p < 0.05; **p < 0.01; and ***p < 0.001.

Model 3 adds JC based on model 2, the coefficient of HRMS is reduced to 0.289 (*p* < 0.001); the coefficient of the impact of JC on creativity is 1.052 (*p* < 0.001), *R^2^* increases by 0.142 and is significant at the level of 0.001. Therefore, the impact of JC mediates the effect of HRMS on employee creativity, hypothesis H_4_ was supported.

Model 4 adds CA on this basis. The coefficient of HRMS on creativity is reduced to 0.129 and the value of *p* is greater than 0.05; the coefficient of JC on creativity is reduced to 0.642 (*p* < 0.001); the coefficient of CA is 0.714 (*p* < 0.001); *R^2^* increases by 0.097 and is significant at the level of 0.001. Therefore, the serial mediation of JC and CA completely mediates the impact of HRMS on employee creativity. Hypotheses H_3_ and H_5_ are supported.

To further test the serial mediating effect, the study uses PROCESS 3.4 (Model 6) to test (demonstrated in Table 4). The results show that the total effect size of the impact of HRMS on employee creativity is 0.419; the lower limit of the 95% CI of the Bootstrap test is 0.042, and the upper limit is 0.161; the effect of HRMS on creativity through JC is 0.158, and the lower limit of the 95% CI of the Bootstrap test is 0.086, and the upper limit is 0.250; the effect of HRMS on creativity through CA is 0.160, the lower limit of the 95% CI of the Bootstrap test is 0.085, and the upper limit is 0.214; finally, the effect of HRMS on employee creativity through JC and CA is 0.101, and the lower limit of the 95% CI of the Bootstrap test is 0.042, and the upper limit is 0.161. After adding two mediating variables, the direct effect of HRMS on employee creativity is not significant.

**Table 4:** The serial mediating effect test.

		Coefficient	BootstapLLCI	BootstapULCI
Direct effect		0.129	−0.008	0.266
	HRS → JC → C	0.158	0.086	0.250
Indirect effect	HRS → CA → C	0.160	0.085	0.214
	HRS → JC → CA → C	0.101	0.042	0.161
Total effect		0.419	0.042	0.161

## Study 2: The Moderating Role of Proactive Personality on Career Construction

### Literature Review and Research Hypothesis

According to CCT, individuals need to develop two meta-competencies in order to achieve career success: self-awareness and adaptability (Akkermans et al., 2020), the individual needs to accommodate the disequilibrium by changing context as well as self (Savickas and Porfeli, 2012). Career personality casts light on the realization of professional self-concept, provides a subjective, individual, and specific perspective of understanding the career, and complements the objective perspective by inspiring and explaining the individual’s subjective self-concepts (Savickas, 2005). Proactive personality is an inescapable embodiment of intrinsic motivation (Seibert et al., 2001), which prompts individuals to manipulate the environment and maximize resources (Oldham and Cummings, 1996), so that affects the role of contextual factors on career outcomes (Shin and Zhou, 2003; Hirst et al., 2009). Employees with highly proactive personalities tend to be self-starters, future-focused and change-oriented (Kim, 2019) and are apt to identify new ways to improve their job performance (Li et al., 2020), which might support creative outcomes. Besides, proactive personality reflects the individual’s tendency towards active change, opportunities hunt, and external environment shaping, and its right synergy will be amplifying the past preoccupations and current aspirations in one’s career building (Savickas and Porfeli, 2012).

First, this feature will tighten the grip of work tasks and resources by employees (Seibert et al., 2001; Li et al., 2014), showing the initiative to look for opportunities, take actions, and persevere with changing the environment by individual efforts (Bateman and Crant, 1993). Second, proactive individuals are willing to innately update knowledge and skills (Li et al., 2020), as well as actively understand the goals of HR practices, in turn, facilitate the development of individual career-related variables, such as CA and creativity (Seibert et al., 2001). Third, employees with remarkable proactive personality have more passion toward work and enjoy more challenges, so they can get a sense of excitement from their work activities and complete these tasks without external control or constraints. Interests triggered by JC can be a catalyst for boosting work behaviors (van de Riet et al., 2015). In all, the control, understanding, and passion boosted by the proactive personality are ingredients of performance improvement, problem-solving, and work advancement (Seibert et al., 2001). Existing research also demonstrates that proactive personality can transform the work by making constructive efforts (Parker and Collins, 2010), negotiating idiosyncratic deals (including flexible scheduling of work hours and special opportunities for skills and career development; Hornung et al., 2008), and taking career initiatives (Seibert et al., 2001).

We demonstrate that proactive personality will attenuate the positive impact of HRMS on employees’ behaviors. The strong intrinsic motivation of proactive personality will undermine the motivational effects of extrinsic motivation (i.e., the HRMS; Ryan and Deci, 2000); traits associated with extrinsic motivation will divert attention away from opportunities for creativity (van Knippenberg and Hirst, 2020). Since proactive individuals are not just passively constructed by situational factors (Pan et al., 2018), instead, they always tend to actively overcome situational constraints (Bateman and Crant, 1993; Kim et al., 2018), they do so by searching for new and more efficient ways of doing things in an effort to improve their performance and demonstrate their creative nature (Alikaj et al., 2021). In this strong context, employees with proactive personalities might be reluctant to risk trying new things despite their personalities because they are compelled to fit in the group (Kim, 2019). In the context of low HRMS, as organizational uncertainty and ambiguity increase, ambiguity sharpens the independence of employees in manipulating and expressing their personal means when assuming work roles (Seibert et al., 1999), individuals can view such ambiguities as opportunities for constructive change that capitalize on their personal strengths, craft a fit between their strengths and job responsibilities (Yi-Feng Chen et al., 2021) and are more likely to behave in idiosyncratic ways (Kim, 2019). A weak situation amplifies the effectiveness of creativity because it can provide clues that lead to a behavioral expression, which is consistent with basic personal tendencies, such as competency and adaptabilities (Yang et al., 2019). Therefore, proactive personality plays a negative moderating role in the process of HRMS on career construction, so the following hypothesis is proposed:

*H5: Proactive personality negatively moderates the relationship of human resource management strength on job crafting and career adaptability.*

### Materials and Methods

Survey questionnaires are from the Chinese financial industry. It mainly explores how the interaction between HRMS as a contextual factor and proactive personality as an individual factor affects employee creativity, and repetitively verifies the mediating role of JC and *CA.*

#### Data Collection

The subjects of the study mainly included front-line service employees, such as sales personnel selling products and services from security companies, tellers from commercial banks, and sales agents from insurance companies. Before distributing the questionnaire, the researcher first contacted subjects to confirm their willingness to participate in the survey, and then the paper questionnaires were handed out and received by mail. A total of 450 questionnaires were distributed to 100 financial companies, and 390 valid questionnaires were returned, with an effective response rate of 86.7%. Among the valid survey samples, in terms of gender, males accounted for 47.6% and females 52.4%.

#### Measures

The measurement of HRMS, JC, CA, and creativity are all carried out on the same scale as in Study 1. The proactive personality comes from existing research and development scales. According to existing research tests, the validity of each scale is good.

##### Proactive Personality

The study adopts the scale developed by Li et al. (2014). The scale includes 10 items, for example, “I am constantly looking for new ways to improve my life,” “I hope to solve problems that will cause other troubles.” The responses for all items were obtained on a five-point Likert scale (1 = completely disagree, 5 = completely agree). Cronbach’s *α* is 0.914.

#### Measurement Models

We use CFA to test the possible common method variance of the main variables (HRMS, JC, CA, creativity, and proactive personality). The results are shown in Table 5. The degree of fitting of five-factor model (χ^2^/*df* = 4.192, RMSEA = 0.084, NFI = 0.980, RFI = 0.940, IFI = 0.985, CFI = 0.984, and GFI = 0.953) is better than other alternative models. In summary, the results measured by this scale are relatively ideal. There is no significant common method variance in this study.

**Table 5:** Confirmatory factor analysis.

Model	*χ*^2^/*df*	RMSEA	NFI	RFI	IFI	CFI	GFI
One-factor model (HRS + JC + CA + C + PP)	29.578	0.252	0.744	0.574	0.751	0.749	0.582
Two-factor model (HRS + JC; CA + C + PP)	17.738	0.193	0.881	0.744	0.887	0.886	0.755
Three-factor model (HRS + JC; CA + C; PP)	6.276	0.108	0.988	0.910	0.990	0.990	0.923
Four-factor model (HRS; JC; CA; C + PP)	4.780	0.092	0.982	0.931	0.985	0.985	0.945
Five-factor model (HRS; JC; CA; C; PP)	4.192	0.084	0.980	0.940	0.985	0.984	0.953

### Results

#### Correlation Analysis

Pearson’s correlation analysis was used to test the correlation between the variables, and the results are shown in Table 6.

**Table 6:** Correlation analysis.

Variables	Mean	S*D*	1	2	3	4	5	6	7	8
1. Gender	1.524	0.500	1							
2. Age	3.980	0.777	0.044	1						
3. Tenure	3.815	1.139	−0.041	−0.673^**^	1					
4. Job level	3.263	0.906	0.080	0.363^**^	−0.375^**^	1				
5. HRMS	3.157	0.393	−0.080	−0.025	0.036	−0.002	1			
6. CA	4.054	0.646	−0.029	0.082	−0.057	−0.064	0.284^**^	1		
7. Creativity	3.713	0.715	−0.093^*^	0.006	0.017	−0.110^*^	0.302^***^	0.585^***^	1	
8. Proactive personality	3.668	0.658	−0.023	0.043	−0.069	−0.070	0.412^***^	0.690^***^	0.672^***^	1
9. JC	3.564	0.501	−0.091	−0.035	0.013	−0.077	0.441^***^	0.668^***^	0.636^***^	0.708^***^

*p < 0.05; **p < 0.01; and ***p < 0.001.

It can be seen from Table 6 that HRMS is positively correlated to CA (*r* = 0.284, *p* < 0.001); creativity is related to gender; the creativity level of female is lower than male (*r* = −0.093, *p* < 0.05); creativity and job level are related; the higher the job level, the stronger the creativity (*r* = −0.110, *p* < 0.05); creativity is positively correlated to HRMS (*r* = 0.302, *p* < 0.001) and CA (*r* = 0.585, *p* < 0.001); proactive personality is positively correlated to HRMS (*r* = 0.412, *p* < 0.001), CA (*r* = 0.690, *p* < 0.001), and creativity (*r* = 0.672, *p* < 0.001); JC is positively correlated to HRMS (*r* = 0.441, *p* < 0.001), creaticity (*r* = 0.668, *p* < 0.001), proactive personality (*r* = 0.636, *p* < 0.001), and JC (*r* = 0.708, *p* < 0.001). This result is the basis for subsequent hypothesis testing.

#### Hypothesis Test

##### The Test of the Main Effect and the Mediating Effect

To test the impact path of HRMS on employee creativity through the serial mediation of JC and CA, all analyses were carried out using SPSS 22.0 to conduct a hierarchical regression analysis, the results are shown in Table 7. The models use employee creativity as the dependent variable; models 5–6 add control variables and HRMS. The results show that the model *R^2^* increases by 0.114 after adding HRMS and is significant at the level of 0.001, HRMS significantly positively affects the level of creativity (*β* = 0.590, *p* < 0.001).

**Table 7:** Main effect and mediating effect test.

	Model 5	Model 6	Model 7	Model 8
Intercept	3.868^∗∗∗^	1.703^∗∗∗^	0.510^∗∗∗^	0.252^∗∗∗^
Gender	−0.120	−0.090	−0.106	−0.091
Age	0.053	0.084	0.049	0.022
Tenure	0.020	0.052	0.019	0.023
Job level	−0.082	−0.079	−0.046	−0.034
HRMS		0.590^∗∗∗^	0.076	0.068
JC			0.844^∗∗∗^	0.558^∗∗∗^
CA				0.330^∗∗∗^
*R^2^*	0.020	0.114	0.409	0.460
*R^2^*	0.020	0.094	0.295	0.051
*F*	1.855	9.362^∗∗∗^	41.955^∗∗∗^	44.109^∗∗∗^

***p < 0.001.

Model 7 adds JC based on model 6, the coefficient of HRMS is reduced to 0.076 (*p* > 0.05); the coefficient of the impact of JC on creativity is 0.844 (*p* < 0.001), *R^2^* increases by 0.295 and is significant at the level of 0.001. Therefore, the impact of JC mediates the effect of HRMS on employee creativity.

Model 8 adds CA on this basis. The coefficient of HRMS on creativity is reduced to 0.068 and the value of *p* is greater than 0.05; the coefficient of JC on creativity is reduced to 0.558 (*p* < 0.001); the coefficient of CA is 0.330 (*p* < 0.001); and *R^2^* increases by 0.051 and is significant at the level of 0.001. Therefore, the serial mediation of JC and CA completely mediates the impact of HRMS on employee creativity. Hypotheses H_3_, H_4_, and H_5_ are confirmed again.

##### The Test of Moderating Effect

**Figure 2:**
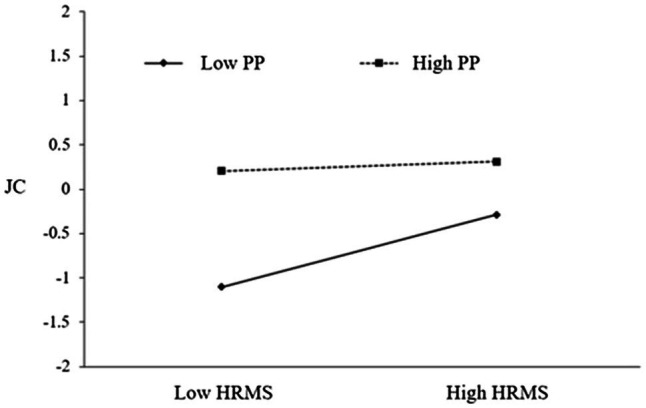
The moderating role of proactive personality in the influence of HRMS on JC.

**Figure 3:**
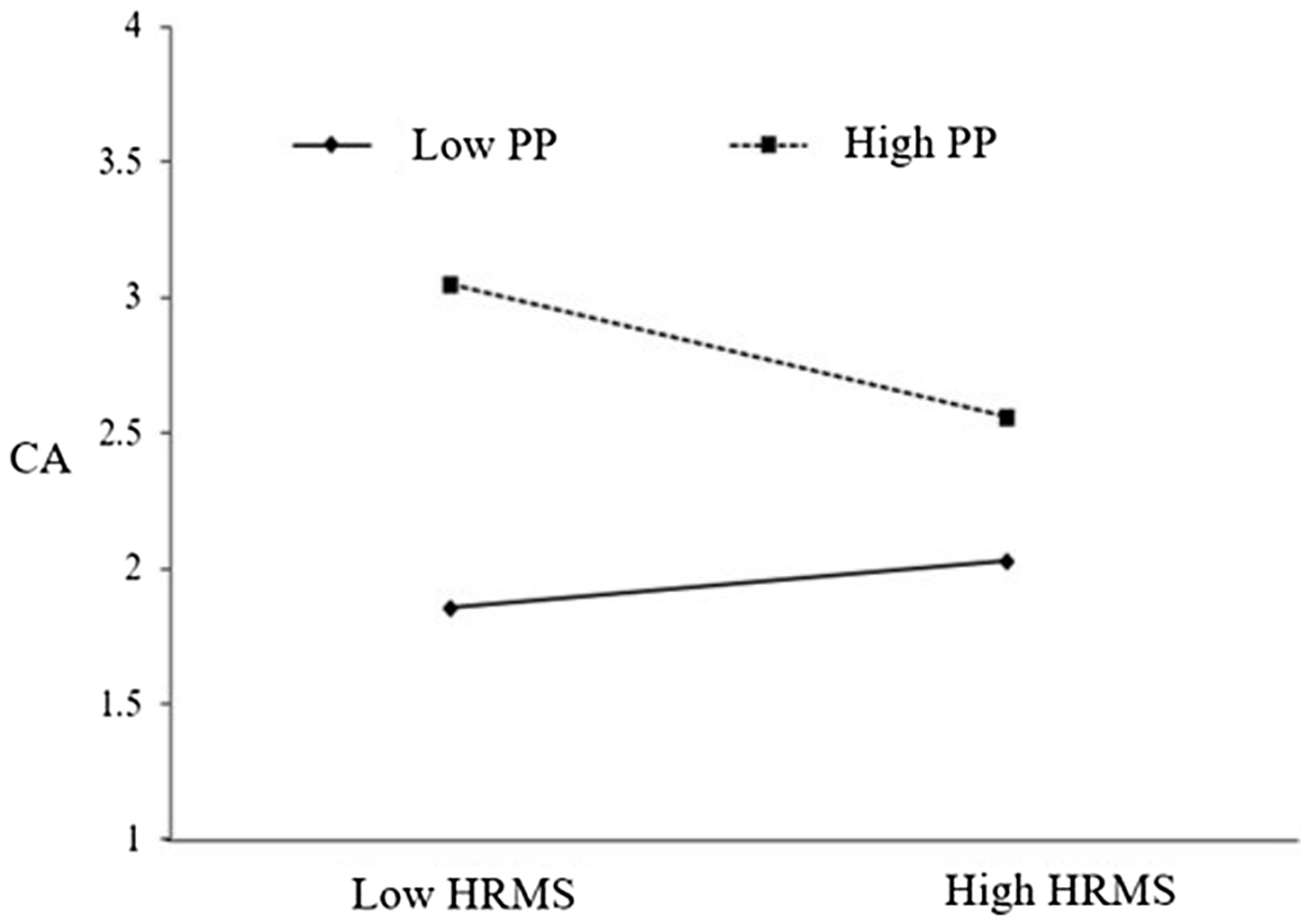
The moderating role of proactive personality in the influence of HRMS on CA.

The moderating effect of proactive personality in the process of HRMS influence on career construction is shown in Figures 2, 3. When the proactive personality is low (−1*SD*), the effect of HRMS on JC is 0.347; when it is high (+1*SD*), the effect size is reduced to 0.114, indicating that over high proactive personality will inhibit the positive effect of HRMS. When the proactive personality is low (−1*SD*), the effect of HRMS on CA is 0.029; when it is high (+1*SD*), the effect size is reduced to −0.189, indicating that higher proactive personality will inhibit the positive impact of HRMS on CA, and even shows a negative impact.

Then we use PROCESS3.4 (Model 84) to test the moderating effect of proactive personality, and the results are shown in Table 8. First, in the impact of HRMS on creativity through JC, the index of the interaction of HRMS and proactive personality is −0.100, the bootstrap 95% CI was −0.171 to −0.010. In the impact of HRMS on creativity through CA, the index of the interaction of HRMS and proactive personality is −0.052, the bootstrap 95% CI was −0.101 to −0.000. In the impact of HRMS on creativity through JC and CA, the index of the interaction of HRMS and proactive personality is −0.028, the bootstrap 95% CI was −0.052 to −0.002. The proactive personality negatively moderates the influence process of HRMS on employee creativity.

**Table 8:** The moderating effect test.

	Variables	Index	LLCI	UUCI
HRMS-JC-C	HRMS*Proactive personality	−0.100	−0.171	−0.010
HRMS-CA-C	HRMS*Proactive personality	−0.052	−0.101	0.000
HRMS-JC-CA-C	HRMS*Proactive personality	−0.028	−0.052	−0.002

## Discussion

The current paper focuses on the joint consideration of actor and contextual factors influencing creativity. The increasing threats of unemployment, higher demand for tasks and resources, and accelerating job complexity have also put forward higher requirements on employees’ CA (Maggiori et al., 2013). Based on previous research, the paper first studied the process of organizational context influencing employee creativity, then clarified the mediating effect of JC and CA in the above-mentioned influence, finally explored the boundary condition, the moderating role of proactive personality, in the process of career construction. Based on CCT, the paper is designed to determine the integrating impact model of HRMS (contextual factors) and proactive personality (individual factors) through JC and CA on employee creativity. Study 1 is based on 297 multi-wave questionnaire data, and Study 2 is based on 390 questionnaire survey data, and the following conclusions are reached:

First, HRMS is an important antecedent of employee creativity. This has been referred to as providing a characteristic and culture of HR practices where employees feel psychologically safe such that blame or punishment will not be assigned for new ideas or breaking with the status quo (Shalley and Gilson, 2004). Second, the ability of long-term career planning process (Klehe et al., 2011), that is CA, mediates the impact of HRMS on creativity through intensifying the immediate investment of human capital and communication in JC (Chen et al., 2007; Ostroff and Bowen, 2016). JC is the adjustment of employees’ work resources and needs, which contributes to shaping an individual’s adaptability and development (Akkermans and Tims, 2017), helping individuals to identify and cope with the difficulties and challenges in their career transitions, achieve better employment (Guan et al., 2015). Third, proactive personality plays a negative moderating role in the process of HRMS on JC and CA, mainly because proactive personality indicates a strong individual intrinsic motivation and weakens the incentive function of HRMS as an extrinsic motivation (Ryan and Deci, 2000). Adaptation is motivated and guided by the goal of bringing inner needs and outer opportunities into harmony (Savickas and Porfeli, 2012). That is, proactive individuals are not only passive recipients of HR policies (Pan et al., 2018), but also are more willing to actively overcome contextual constraints (Bateman and Crant, 1993), rather than being constrained, constructed, and changed by the environment (Chen et al., 2020). Therefore, with the increase of proactive personality, the positive effects of HRMS on JC and CA have been inhibited.

This research supports the interactive effects of contextual factors and individual characteristics on career construction, as well as the utility of career-ability to distal employee creativity. The findings make several contributions to the current literature.

First, we discussed employee creativity through the entire career construction process to ensure the integrity of the career construction research (Chen et al., 2020). Similar to career competencies, career adaptability is a malleable resource that allows individuals to solve complex problems throughout their careers in a relatively long life-span (Super, 1980; Savickas, 1997; Akkermans et al., 2020). Nevertheless, the previous research emphasized the effect of career readiness (personality trait of the flexibility of willingness to change) when arguing the shaping of CA (Savickas, 2005; Savickas and Porfeli, 2012), current research draws attention to repeated career behaviors (JC) in the formation of *CA.* To develop these adaptive-abilities, individuals need to continuously gain insights into their own characteristics and the complexity of working environments through various personal exploring and crafting experiences (Cai et al., 2015; Guan et al., 2015). Although most of the research on career construction focuses on the sequential model of adaptivity-adaptability-adapting-adaptation (Pan et al., 2018), where JC is the outcome of *CA.* We take employees JC as an antecedent variable that affects CA, in view that the two are linked and integrated. We assess CA from life-span perspective—from short-term fluctuations in vocational experiences and behavior to career development and construction over the adult lifespan (Savickas, 1997). That is, CA can be trained and thus it is indeed a learnable competence (Mei et al., 2021). Vogt et al. (2015) verified the reverse causality through longitudinal research and argued that JC can help establish an individual’s psychological resources, while the level of psychological resources cannot significantly predict future job reshaping behaviors.

Second, we provide a complex and reasonable explanation for the interaction within contextual factors, individual factors, and career-related psychosocial resources. This shift in attention from the individual focus and the creative few toward the contextual view and then toward the integrative view (Joo et al., 2013) coincides with contextual and multicultural perspectives on work (Savickas, 1997). There are various HR practices that have a constructive role in the development of individual competencies and are essential for innovative behavior (Yasir and Majid, 2020). While changing managerial situations alone will not help creativity, the HRM system should be delivered and applied in a concerted way with a holistic perspective (Joo et al., 2013), incorporating elements of employees creative behaviors and traits. Previous research has suggested that HR practices are linked to employees’ creative work behavior through different mechanism (Rehman et al., 2019), so the current paper pays attention to the mediating effect of JC and *CA.* Career construction not only enables employees to take responsibility for their work behaviors by showing self-discipline but also helps them translate HRMS into meaningful creative behaviors (Lan and Chen, 2020). Furthermore, this paper expands the border of creativity literature. We found that individual ability was an important antecedent of creativity beyond affection, cognition, training, personality, and social environment (Hennessey and Amabile, 2010), especially under the background of boundaryless careers.

Finally, we highlight the interactionist perspective as an integrative theoretical lens for developing an in-depth understanding of antecedents of creativity in the workplace (Zhou and Hoever, 2014). The career construction process is affected by proactive personality (willingness or readiness to affect change), which manifests in varying states of activation (Savickas and Porfeli, 2012). More importantly, it is an intriguing finding that the moderating effect of proactive personality is negative, which supports existing research (George and Zhou, 2001; De Dreu and Weingart, 2003; Jimmieson et al., 2004; Spychala and Sonnentag, 2011), challenging the positive effects of proactive personality (Oldham and Cummings, 1996; Seibert et al., 2001; Hornung et al., 2008; Parker and Collins, 2010; Li et al., 2014), and helping understand the duality of proactive personality. Although with stronger ability and motivation, creativity may be more likely regardless of the situation, when HRMS and proactive personality are (potentially) positive, the result is a pattern that can be described as diminishing gains (Zhou and Hoever, 2014), that is, individuals in lack of trait-based motivation may be more creative when operating in an environment with greater and more explicit expectations for creativity (van Knippenberg and Hirst, 2020). Proactive personality leads to more network-style construction activities for individuals, thereby reducing their dependence on the environment (Parker and Collins, 2010; Pan et al., 2018). The strong willingness of employees to redesign their jobs is caused by the intrinsic motivation of proactive personality, rather than the extrinsic motivation provided by HRMS (Wang et al., 2017; Federici et al., 2021).

### Limitations and Future Research

There are still some limitations in the design and implementation of the empirical research. First, the research adopted multi-wave questionnaires in data processing, and collected data at three points in time, which minimized the problem of common method variance, but the verification of causality was still not sufficient. In future research, researchers can use multi-source and time data, longitudinal research, and experimental design to alleviate the above problems. Second, current research did not consider the impact of possible changes in personality traits, JC, and CA over time. Some existing studies believe that personality traits will be moderately extended over time (Li et al., 2014). CA is a psychological resource generated by accumulation, and it will also undergo plastic changes over time. Similarly, JC is also a process in which individuals generate and create changes over time (Wrzesniewski and Dutton, 2001; Bruning and Campion, 2018). These possible changes over time may have an impact on the research conclusions. Future research can use other data processing methods such as the potential growth model to explore the dynamic impact effects of these variables in a longer period of time and under specific circumstances (Johnston, 2018).

### Practical Implication

This research also has practical implications for managers to promote the career construction process of employees through the improvement of the HR system, and then enhance the creative performance of employees and organizations. First, organizations can deepen the overall understanding and recognition of organizational policies among managers and employees by strengthening the organizational context factor of HRMS, enhancing employees’ perception of leadership support and communication effectiveness, ensuring a safe working environment, promoting employees’ JC and CA accumulation, and then tap into employee creativity. In addition, in the current working environment affected by the epidemic and the rapid development of science and technology, the relationship between the organizational context and the individual characteristics of employees should be reconciled to promote the compatibility and complementation between the people and environment, as well as steer clear of wasting resources due to being over-enthusiastic in a certain aspect, refraining from putting the cart before the horse. Finally, the CA of employees is a long-term development process, which is formed by the continuous accumulation of JC, and the purpose of JC is to achieve the consistency and compatibility of the resources provided by the organization with its own job roles and task requirements. Therefore, the resources should be constantly adjusted by organizations to motivate employees to redesign their jobs in the short term, thereby translating into CA as a psychosocial resource and distal creative behaviors.

## Data Availability Statement

The raw data supporting the conclusions of this article will be made available by the authors, without undue reservation.

## Author Contributions

JZ: conceptualization, formal analysis, methodology, and writing—original draft. FZ: funding acquisition, investigation, project administration, supervision, validation, and writing—review and editing. NL: data curation and writing—review and editing. ZC: validation, supervision, writing—review and editing, and validation. All authors contributed to the article and approved the submitted version.

## Funding

This research was funded by key project of Beijing Social Science Foundation “The cross-layer influence and theoretical construction of the employer brand on the behavior and performance of service employees in Beijing service companies” (19GLA009).

## Conflict of Interest

The authors declare that the research was conducted in the absence of any commercial or financial relationships that could be construed as a potential conflict of interest.

## Publisher’s Note

All claims expressed in this article are solely those of the authors and do not necessarily represent those of their affiliated organizations, or those of the publisher, the editors and the reviewers. Any product that may be evaluated in this article, or claim that may be made by its manufacturer, is not guaranteed or endorsed by the publisher.
